# Improved gene editing and fluorescent-protein tagging in *Aspergillus nidulans* using a Golden Gate-based CRISPR-Cas9 plasmid system

**DOI:** 10.12688/wellcomeopenres.23086.1

**Published:** 2024-10-17

**Authors:** Domenico Modaffari, Aimée Finlayson, Yuyang Miao, Edward W. J. Wallace, Kenneth E. Sawin

**Affiliations:** 1 Wellcome Centre for Cell Biology and Institute of Cell Biology, School of Biological Sciences, Wellcome Centre for Cell Biology and Institute of Cell Biology, School of Biological Sciences, University of Edinburgh, Michael Swann Building, Max Born Crescent, Edinburgh, EH9 3BF, UK; 2 Institute for Cell Biology and Centre for Engineering Biology, School of Biological Sciences, University of Edinburgh, C.H. Waddington Building, Max Born Crescent, Edinburgh, Scotland, EH9 3BF, UK

**Keywords:** CRISPR-Cas9, Aspergillus, Fluorescent Protein, Cell Biology

## Abstract

CRISPR-Cas9 systems can be used for precise genome editing in filamentous fungi, including *Aspergillus nidulans*. However, current CRISPR-Cas9 systems for *A. nidulans* rely on relatively complex or multi-step cloning methods to build a plasmid expressing both Cas9 and an sgRNA targeting a genomic locus. In this study we improve on existing plasmid-based CRISPR-Cas9 systems for *Aspergilli* by creating an extremely simple-to-use CRISPR-Cas9 system for *A. nidulans* genome editing. In our system, a plasmid containing both Cas9 and an sgRNA is assembled in a one-step Golden Gate reaction.

We demonstrate precise, scarless genome editing with nucleotide-level DNA substitutions, and we demonstrate markerless gene tagging by fusing fluorescent-protein coding sequences to the endogenous coding sequences of several *A. nidulans* genes. We also describe *A. nidulans* codon-adjusted versions of multiple recent-generation fluorescent proteins, which will be useful to the wider *Aspergillus* community.

## Plain Language Summary

For nearly a century, *Aspergillus nidulans*, a type of mold, has been a key tool for scientists to explore the functions of cells. *Aspergillus nidulans* is non-pathogenic, but related *Aspergillus* species are important human pathogens. Researchers often modify the DNA of *Aspergillus* species to understand more about the function of genes, units of DNA that contain the instructions needed for the cell to make proteins. In the last decade, a new method called CRISPR-Cas9 has revolutionized how scientists edit DNA. CRISPR-Cas9 has been adapted for use in many organisms, including different types of *Aspergillus* species. Building on existing tools, we developed a CRISPR-Cas9 system tailored for *A. nidulans* that is both cheaper and easier to use. We tested our new system by editing several *Aspergillus nidulans* genes. We have also used our system to incorporate recently-developed fluorescent proteins into *Aspergillus nidulans* cells. These proteins glow in the presence of certain wavelengths of light, allowing researchers to easily see and track where specific proteins are within the cell. Our system will be adopted by researchers looking to explore and manipulate genes more effectively in *Aspergillus nidulans*.

## Introduction

The filamentous fungus *Aspergillus nidulans* was first developed as a model system for microbial genetics in the 1940s and 1950s ( Pontecorvo *et al.*, 1953). Since then, *A. nidulans* has served as a platform for numerous important discoveries and insights in cell biology, particularly in cell-cycle regulation, microtubule biology, and vesicular trafficking ( Osmani & Mirabito, 2004; Pinar & Peñalva, 2021). In recent decades, *A. nidulans* has become less commonly used as a model system for cell biology, due largely to the ease of genetic manipulation in other model systems such as yeasts (e.g. *Saccharomyces cerevisiae*, *Schizosaccharomyces pombe*). However, from a biological perspective, *A. nidulans* has much to offer: it is multicellular, more complex than yeasts; it is closely related to the fungal pathogen *Aspergillus fumigatus* (designated a critical priority pathogen by the World Health Organization ( WHO, 2022)); and it produces numerous uncharacterized natural products ( Caesar *et al.*, 2020). Therefore, *A. nidulans* remains an excellent model organism and platform for future discovery research.

Targeted molecular-genetic modifications in *A. nidulans* are often made via homologous recombination of a DNA cassette containing long (>1 kb) homology arms. Such a cassette is typically produced via fusion PCR ( Szewczyk *et al.*, 2006) and normally includes a selectable marker as well as the genetic modification of interest. The number of available selectable markers in *A. nidulans* can limit the number of modifications possible in a given strain. This issue can be addressed in part by using larger collections of markers ( Dohn *et al.*, 2018), but such an approach also requires the use of *A. nidulans* strains with multiple auxotrophies. Alternatively, the commonly-used *pyrG* marker (encoding orotidine 5’ phosphate decarboxylase) can be used for targeting multiple times, as it can be recycled through successive rounds of positive and negative selection; however, this is a lengthy procedure ( Nielsen *et al.*, 2006). Overall, neither of these approaches to genetic modification is ideal, and commonly-used techniques such as gene tagging and gene deletion typically take longer in *A. nidulans* than in yeasts ( Todd *et al.*, 2007a; Todd *et al.*, 2007b).

The CRISPR-Cas9 system ( Jinek *et al.*, 2012) provides an attractive alternative to the gene-targeting approaches described above. CRISPR-Cas9 allows precise genome editing and can operate at sufficiently high efficiency that a selectable marker is no longer required to identify and isolate the genetic modification of interest. Briefly, a single-guide RNA (sgRNA) forms a ribonucleoprotein complex with endonuclease Cas9, which cleaves DNA at a specific genomic-locus “protospacer” sequence, complementary to a 20-nucleotide “spacer” sequence present within the sgRNA ( Jinek *et al.*, 2012). The protospacer must be followed directly by a Protospacer Adjacent Motif (PAM), which for Cas9 is the nucleotide sequence “NGG”. Cas9 then produces a double-stranded break (DSB) in DNA, approximately three nucleotides upstream of the PAM ( Jinek *et al.*, 2012). CRISPR-Cas9 thus allows the induction of a sequence-specific DSB at almost any desired site in the genome, provided that it is adjacent to an NGG trinucleotide. The DSB caused by CRISPR-Cas9 can be exploited to introduce targeted genetic modifications via different DSB repair mechanisms, such as homology-directed repair (HDR) and non-homologous end joining (NHEJ) ( Adli, 2018).

Genetic manipulation via CRISPR-Cas9 has several advantages compared to previous methods. First, genome editing is possible using relatively short homology (<100 bp) arms ( Nødvig *et al.*, 2018; Schuster & Kahmann, 2019; Zhang *et al.*, 2016), making it much easier to generate homology-repair (HR) templates. Second, markerless/scarless editing avoids the need for selectable markers ( Schuster & Kahmann, 2019). Third, markerless gene tagging allows modified genomic loci to retain endogenous promoters, untranslated regions, and terminator elements, thereby preserving both transcriptional and post-transcriptional gene regulation; by contrast, when gene tagging involves a selectable marker, one or more of these elements is typically disrupted. Finally, markerless editing and tagging allow the introduction of an unlimited number of genetic modifications within the same strain.

Since their discovery, CRISPR systems have been used for genetic modification of model and non-model organisms, including *Aspergilli* ( Schuster & Kahmann, 2019). Delivery of CRISPR-Cas9 complexes for genome editing in filamentous fungi is typically achieved either by: 1) expressing the CRISPR-Cas9 complex *in vivo* from an autonomously-replicating plasmid, or 2) reconstituting the CRISPR-Cas9 complex *in vitro* and introducing it into cells ( Schuster & Kahmann, 2019; Shen *et al.*, 2023; Song *et al.*, 2019). The price of reagents makes the reconstitution approach more expensive than plasmid-based CRISPR systems, especially if it is necessary to test multiple sgRNAs for a given locus.

A popular plasmid-based CRISPR-Cas9 system for filamentous fungi, including *Aspergilli,* was developed in the Mortensen group by Nødvig *et al*. ( Nødvig *et al.*, 2018; Nødvig *et al.*, 2015), with more than 250 plasmid requests on Addgene as of July 2024. In this system, the sgRNA spacer is inserted into a Cas9-expressing vector via PCR and USER cloning ( Geu-Flores *et al.*, 2007; Nødvig *et al.*, 2018; Nødvig *et al.*, 2015). While this system has been shown to be highly effective for genome editing, the USER cloning method requires an adenine and a thymine spaced 6 to 15 nucleotides apart within the spacer, which could limit spacer selection ( Nødvig *et al.*, 2018). Furthermore, USER cloning requires modified DNA oligonucleotides that include deoxyuridine ( Nødvig *et al.*, 2018), which increases the price of the reaction (£10 per deoxyuridine, Integrated DNA Technologies, June 2024). Thus, despite the strengths of the system developed by Nødvig *et al.*, it would be desirable to have a simpler method to introduce sgRNA spacer sequences into the system.

Here we build on the system of Nødvig *et al.* ( Nødvig *et al.*, 2018; Nødvig *et al.*, 2015) and incorporate a Golden Gate-based sgRNA cloning approach ( Engler *et al.*, 2008; Torres-Garcia *et al.*, 2020) to construct an efficient, inexpensive, and easy-to-use plasmid-based CRISPR-Cas9 system for use in *A. nidulans*. We show that our system can be used both for precise genome editing and for markerless gene tagging with a range of recent-generation fluorescent proteins.

## Results

### Improving an existing CRISPR system

To improve upon the system of Nødvig *et al.*, we modified their CRISPR-Cas9 vectors to incorporate several features of a CRISPR system developed by Torres-Garcia *et al.* for fission yeast *Schizosaccharomyces pombe* ( Torres-Garcia *et al.*, 2020). Similarities and differences between our approach and that of Nødvig *et al.* are summarized in Table 1; we describe three key differences below.

**Table 1.** Features of the CRISPR plasmid system generated in this study. Differences in our system compared to that of Nødvig *et al.* ( Nødvig *et al.*, 2018) are indicated in bold.

Features	Nødvig *et al.* ( Nødvig *et al.*, 2018)	This study
Fungal maintenance sequence	AMA1	AMA1
Cas9	*Aspergillus niger* codon-adjusted Cas9-NLS driven by *A. nidulans tef1* promoter	*Aspergillus niger* codon-adjusted Cas9-NLS driven by *A. nidulans tef1* promoter
sgRNA transcription	sgRNA driven by *Aspergillus fumigatus* U3 promoter	sgRNA driven by *Aspergillus fumigatus* U3 promoter
sgRNA processing	sgRNA liberated by flanking tRNAs	**sgRNA liberated by HDV ribozyme at 5’ end and tRNA at 3’ end**
Spacer cloning	Insertion of spacer sequence into vector via USER cloning (allows multiple sgRNAs per plasmid)	**Insertion of spacer sequence into vector via Golden Gate cloning (PaqCI).** **Visual selection in bacteria via sfGFP pop-out cassette**
Selectable markers	*pyrG*, ***argB***, *hph*, *ble*	*pyrG*, *hph*, *ble*, ***NAT***

First, we introduce the sgRNA spacer sequence into CRISPR-Cas9 vectors in a one-pot reaction by Golden Gate cloning, using two annealed complementary 60-mer oligonucleotides as the insert ( Torres-Garcia *et al.*, 2020). Golden Gate cloning depends on digestion of both vector and insert with a type IIS restriction enzyme ( Engler *et al.*, 2008), and the recognition sequence for the enzyme must be present in the vector only at the insert cloning site. The type IIS enzyme BsaI, which recognises a 6-base sequence, is most commonly used in Golden Gate cloning and was used by Torres-Garcia *et al.* ( Torres-Garcia *et al.*, 2020); however, we found that the Nødvig *et al.* CRISPR-Cas9 vectors contained 4 or more BsaI sites, depending on the specific vector. Rather than remove these sites, we chose to use a different type IIS enzyme, PaqCI (isoschizomer of AarI), at the Golden Gate cloning site. Because PaqCI has a 7-base rather than a 6-base recognition sequence, this also decreases the likelihood that the enzyme recognition sequence for Golden Gate cloning will be present within a spacer sequence or in any future modified forms of our CRISPR-Cas9 vectors.

**Figure 1.**
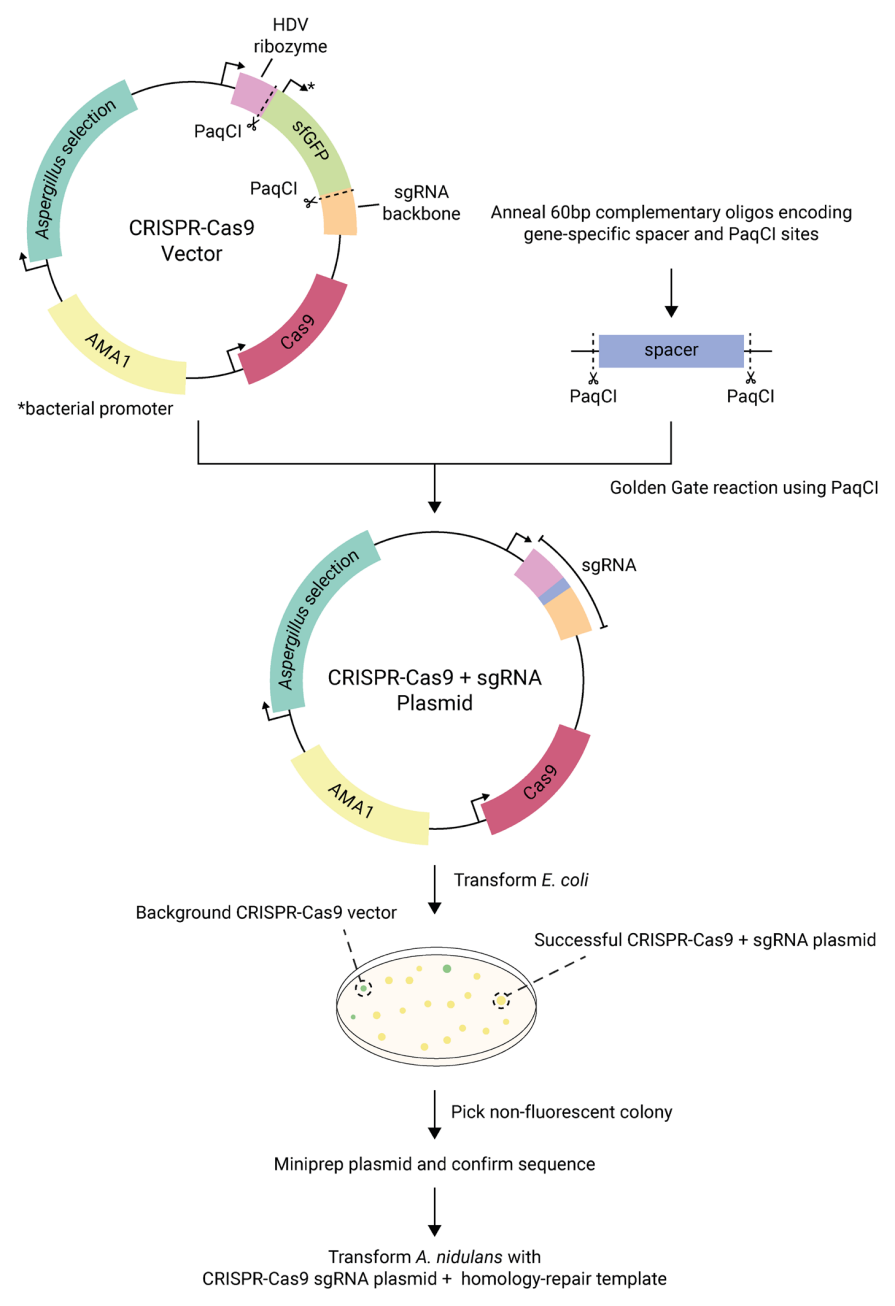
Overview of Golden Gate-based sgRNA plasmid assembly. A CRISPR-Cas9 vector is mixed in a PaqCI Golden Gate reaction with a short dsDNA fragment containing the spacer sequence. The resulting plasmid gains an assembled sgRNA and loses a superfolderGFP (sfGFP) cassette that would otherwise be expressed in bacteria. The plasmids also contain a bacterial replication origin and ampicillin-resistance gene (not shown). Correctly-assembled plasmids can be identified by the absence of green fluorescence/color in the *E. coli* transformant colonies. The plasmid can then be recovered and used for editing in *A. nidulans*.

Second, our CRISPR-Cas9 vectors contain a superfolderGFP (sfGFP) cassette at the prospective spacer insertion site, under the control of a constitutive bacterial promoter ( Figure 1) ( Torres-Garcia *et al.*, 2020). Correct Golden Gate-based insertion of the spacer sequence into the CRISPR-Cas9 vector leads to excision of the sfGFP cassette (and its promoter), allowing simple visual selection of colonies on bacterial transformation plates; incorrect clones appear green on plates, while correct clones are colorless. Altogether, we found our PaqCI-based sgRNA spacer cloning approach to be extremely efficient; in experiments involving cloning more than 50 spacers into our CRISPR-Cas9 vectors, we never found sequence errors after Sanger sequencing.

Third, in our approach, the sgRNA is liberated by an HDV ribozyme at the 5’ end of the spacer sequence ( Torres-Garcia *et al.*, 2020). The HDV remains attached at the 5’ end of the liberated sgRNA, which may increase sgRNA stability ( Ryan *et al.*, 2014).

Finally, to increase the number of dominant selectable markers for *A. nidulans* transformation, we constructed a CRISPR-Cas9 vector containing the nourseothricin resistance gene ( *NAT*) driven by the *A. fumigatus pyrG* promoter and terminator. We found that untransformed protoplasts were sensitive (i.e. could not form colonies) at concentrations of nourseothricin above 100 µg/mL, while protoplasts transformed with the *NAT*-containing CRISPR-Cas9 vector were resistant (Extended data Figure S1). Notably, nourseothricin was effective only when transformation plates contained sucrose (1 M) ( Oakley *et al.*, 2012) as an osmotic balancer. When KCl (0.6 M) was used as an osmotic balancer, untransformed protoplasts were resistant to all concentrations of nourseothricin tested (up to 200 µg/mL; Extended data Figure S1).

### Scarless nucleotide-level gene editing

**Figure 2.**
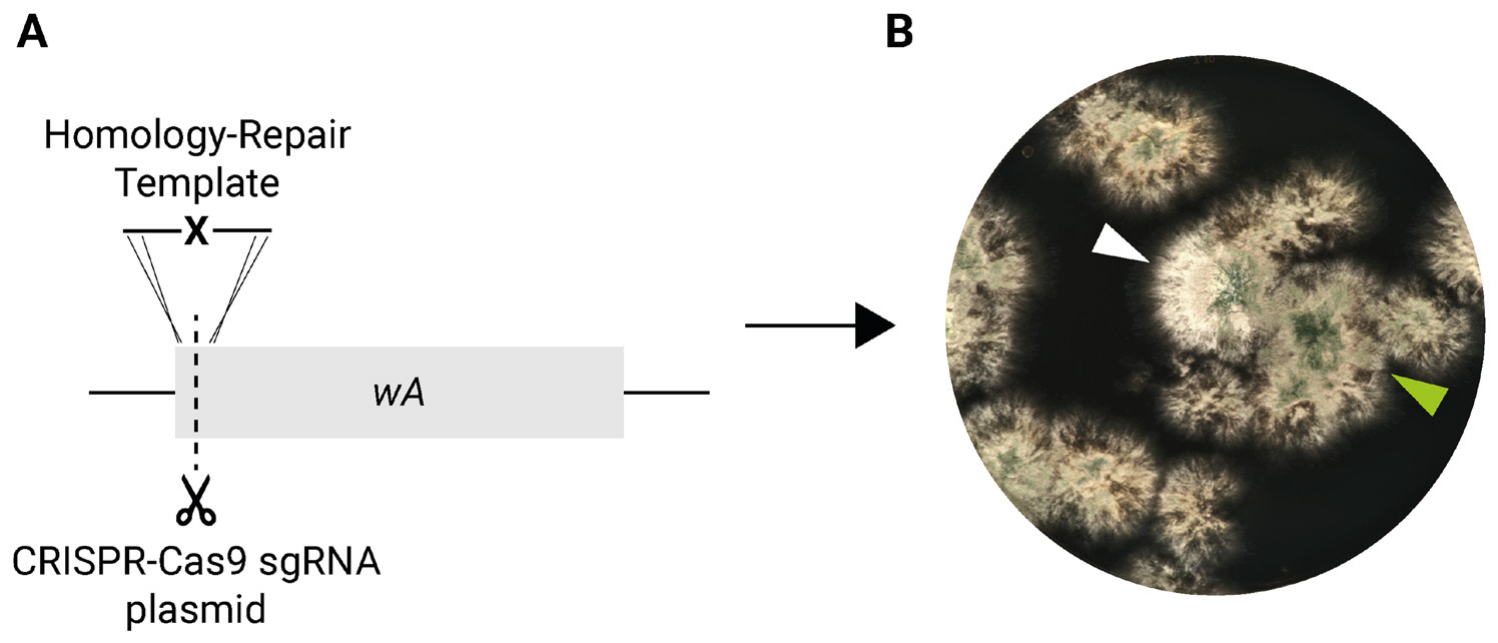
Introduction of an early stop codon mutation in *wA* with CRISPR-Cas9. **A.** Diagram of *wA* gene editing. Dashed line indicates double-stranded break in DNA, induced by Cas9-sgRNA complex expressed from plasmid. ‘X’ in DNA homology-repair template indicates stop codon. **B.** Transformation plate after 3 days at 37°C and 2 days at room temperature. White arrowhead points to a successfully edited colony (white spores). Green arrowhead points to an unedited colony (green spores).

To confirm that our CRISPR-Cas9 plasmids retain the ability to perform genome editing, we initially introduced a stop codon into a gene coding sequence using a single-stranded HR template, as originally tested by Nødvig *et al.* ( Nødvig *et al.*, 2018). We chose the *wA* gene as a target, as disruption of *wA* leads to white-colored conidia, which are easily detected on solid media ( Mayorga & Timberlake, 1992). Previous work has shown that the efficacy of CRISPR in *Aspergilli* can vary, depending on spacer sequence ( Nødvig *et al.*, 2018). In our test to edit *wA*, we found only a minority of colonies with white conidia ( Figure 2; Extended data Table S1). Nevertheless, the intended genomic modification was successful, as verified by Sanger sequencing, and we had similar success using a double-stranded HR template (Extended data Table S1).

Although the *wA* editing was relatively inefficient, we achieved a much higher success rate for nucleotide-level modification in the *sakA* gene (Extended data Table S1). By contrast, similar attempts to modify the *cotA* gene were unsuccessful (Extended data Table S1). Locus-dependent variability of editing efficiency is not surprising, as both spacer sequence and local chromatin state can affect DNA cleavage by Cas9 ( Hsu *et al.*, 2013; Isaac *et al.*, 2016).

Overall, these results indicate that our CRISPR-Cas9 system can be used for scarless genome editing at the level of individual nucleotide bases, although success can depend on the efficiency of the sgRNA-Cas9 at the targeted genomic locus.

### Markerless gene tagging with fluorescent proteins

As *A. nidulans* is a model organism for cell biology, we next tested the utility of our CRISPR-Cas9 system for gene tagging, which is particularly important for microscopy and protein biochemistry. We generated multiple tagging cassettes coding for recent-generation fluorescent proteins (FPs), codon-adjusted for *A. nidulans* ( Table 2), and we used these with our CRISPR-Cas9 system for both N- and C-terminal tagging. As part of our workflow, we purified clonal strains by dissecting and plating individual spores recovered from transformant colonies. This takes less time than repeated streak-purification of transformants, decreasing strain-construction time to 7–10 days.

**Table 2.** *A. nidulans* codon-adjusted fluorescent proteins used in this study.

Fluorescent protein	λ _ex_ / λ _em_ (nm)	Origin
mStayGold	499/510	Ando *et al.* ( Ando *et al.*, 2024)
mStayGold2	499/509	Ando *et al.* ( Ando *et al.*, 2024)
mNeonGreen	506/517	Shaner *et al.* ( Shaner *et al.*, 2013)
mScarlet3	569/572	Gadella *et al.* ( Gadella *et al.*, 2023)
mCardinal	604/659	Chu *et al.* ( Chu *et al.*, 2014)

λ _ex_ = maximum excitation wavelength λ _em_ = maximum emission wavelength

**Figure 3.**
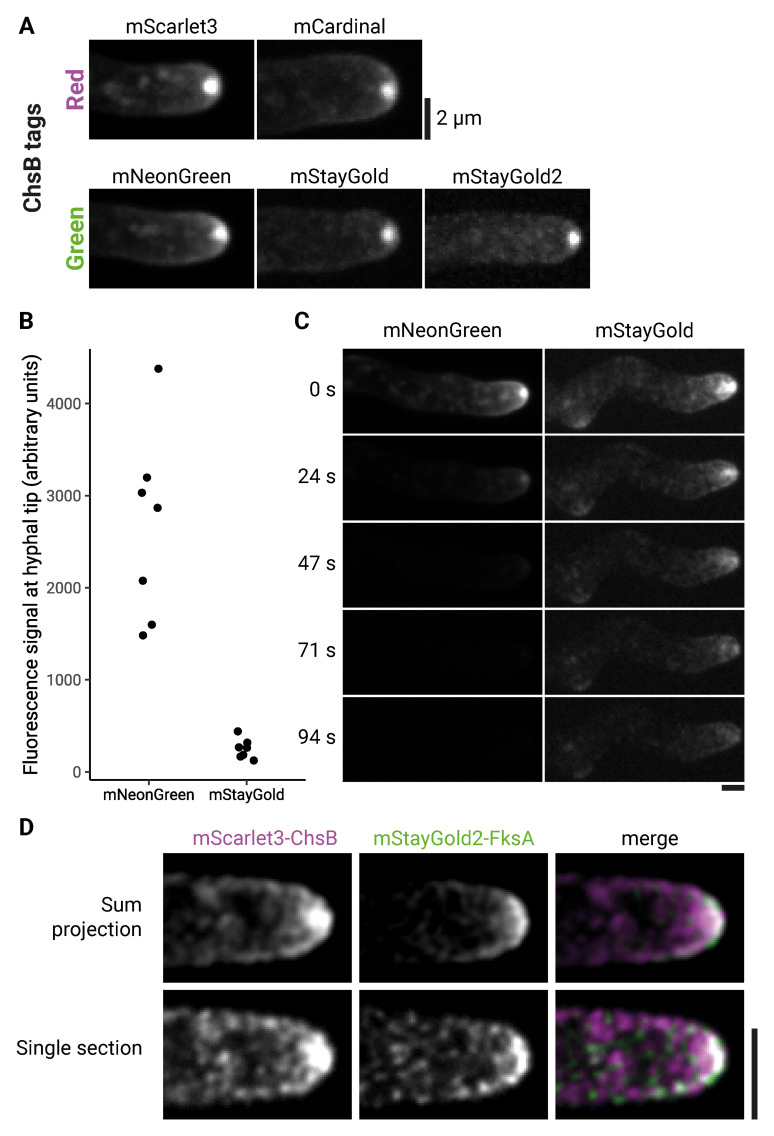
Markerless fluorescent-protein tagging with CRISPR. **A.** Maximum intensity projections of chitin synthase B (ChsB) tagged at its N-terminus with the fluorescent proteins mScarlet3 (strain aDM018), mCardinal (strain aDM048), mNeonGreen (strain aDM045), mStayGold (strain aDM046), and mStayGold2 (strain aDM047). **B.** Quantification of hyphal tip fluorescence for mNeonGreen-ChsB (aDM045) and mStayGold-ChsB (aDM046). p < 0.05 by Welch’s two-tailed t-test. n = 7 for both strains. **C.** Photostability of mNeonGreen-ChsB (aDM045) and mStayGold-ChsB (aDM046). Images are individual timepoints (seconds) from a movie acquired with continuous laser exposure. Micrographs are sum intensity projections. Contrast settings were adjusted separately for each strain (to give comparable appearance at 0 sec) but are the same for all timepoints within the same strain. **D.** Micrographs of mScarlet3-ChsB, mStayGold2-FksA (1,3-beta-glucan synthase) double-tagged strain (aDM029). Scale bars = 2µm.

We fused the coding sequence of each of the FPs mNeonGreen, mCardinal, mScarlet3, mStayGold, and mStayGold2 ( Table 2) to the 5’ end of the coding sequence of transmembrane protein chitin synthase B (ChsB), at the endogenous *chsB* locus ( Figure 3A). All the FP-tagged ChsB fusion proteins were visible by fluorescence microscopy, and all localized to the expected location—the inner layer of the spitzenkörper (SPK), a structure containing small vesicles, cytoskeletal elements and ribosomes, in close apposition to the fungal hyphal tip ( Fukuda *et al.*, 2009; Steinberg *et al.*, 2017). In these experiments, we found that tagging could be successful using homology arms as short as 30 bp; in other experiments, involving N- or C-terminal tagging of six other genes, we found that tagging could be successful using homology arms as short as 20 bp (Extended data Table S2).

Among the FPs we tested, we had a particular interest in the newly-developed FP mStayGold, which has been described to be reasonably bright and very photostable, at least when expressed in mammalian cells ( Ando *et al.*, 2024). Surprisingly, we found that mStayGold-ChsB was approximately ten times less bright than mNeonGreen-ChsB ( Figure 3B). While we do not currently have an explanation for the low fluorescence of mStayGold-ChsB, in this instance it was sufficiently bright for imaging, presumably because of the normally high expression level of ChsB. Consistent with work in mammalian cells ( Ando *et al.*, 2024), in time-lapse imaging experiments we found that mStayGold-ChsB was substantially more photostable than mNeonGreen-ChsB ( Figure 3C).

### Successive rounds of tagging

We next tested how well our CRISPR-Cas9 system could work for multiple rounds of gene tagging, as tagging two or more different proteins in the same cell is often critical for determining protein colocalization and/or protein-protein interactions. Starting from an mScarlet3-ChsB strain, we tagged the N-terminus of transmembrane protein glucan synthase FksA with mStayGold2. To our knowledge, FksA has not been tagged previously in *A. nidulans*. In these experiments, we used a CRISPR-Cas9 plasmid containing the same selectable marker ( *pyrG*) for both rounds of tagging, as *A. nidulans* readily loses plasmids in the absence of selection ( Gems *et al.*, 1991).

We then used the doubly-tagged strain to investigate FksA localization relative to ChsB. Like mScarlet3-ChsB, mStayGold2-FksA localized to hyphal tips ( Figure 3D). However, examination of individual Z-sections showed that the two proteins did not fully co-localize; rather, mStayGold2-FksA appeared to localize to regions immediately surrounding or adjacent to mScarlet3-ChsB ( Figure 3D). In *Neurospora crassa,* glucan synthase FKS-1 (homolog of *A. nidulans* FksA) similarly localizes adjacently to chitin synthase CHS-1 ( Sánchez-León & Riquelme, 2015). Our results thus confirm that the stratification of the SPK seen in *N. crassa* is conserved in *A. nidulans*, as was first shown by Schultzhaus *et al.* on the basis of the localization of other SPK-associated proteins ( Peñalva, 2015; Schultzhaus *et al.*, 2015).

In summary, our CRISPR-Cas9 system allowed us to successively tag ChsB and FksA at their endogenous loci in a way that is both simpler and quicker than traditional methods.

## Discussion

Here we report an improved CRISPR-Cas9 method for genetic manipulation of *A. nidulans*; we anticipate that this will be a powerful tool for cell biology research. We modified an existing plasmid-based CRISPR system for filamentous fungi ( Nødvig *et al.*, 2018) to enable fast, efficient and inexpensive cloning of an sgRNA spacer sequence into a CRISPR-Cas9 vector available with a variety of selectable markers ( Figure 1). Most notably, inserting spacer sequences via Golden Gate cloning greatly facilitates the testing of multiple sgRNAs for one or more loci. We also added another dominant selection marker to the available CRISPR-Cas9 vectors. This increases versatility and may allow simultaneous transformation with multiple plasmids. We show that our CRISPR-Cas9 system can be used for easy, precise gene editing and gene tagging, using short homology arms ( Figure 2, Figure 3; Extended data Tables S1, S2). Overall, our CRISPR-Cas9 system can be used for most single-locus genetic modifications in *A. nidulans*. All vectors and FP cassettes are available via Addgene ( Table 3).

**Table 3.** Plasmids shared on Addgene.

Identifier	Addgene number	Use
pDM026	216808	Vector for Aspergillus CRISPR-Cas9 genetic engineering with Golden Gate cloning drop-out cassette for spacer insertion and pyrG selectable marker.
pDM028	216809	Vector for Aspergillus CRISPR-Cas9 genetic engineering with Golden Gate cloning drop-out cassette for spacer insertion and hph selectable marker.
pDM030	216810	Vector for Aspergillus CRISPR-Cas9 genetic engineering with Golden Gate cloning drop-out cassette for spacer insertion and ble selectable marker.
pDM068	216811	Vector for Aspergillus CRISPR-Cas9 genetic engineering with Golden Gate cloning drop-out cassette for spacer insertion and NAT selectable marker.
pDM042	216812	*Aspergillus nidulans* codon-adjusted mScarlet3 fluorescent protein, includes linker for C-terminal tagging.
pDM043	216813	*Aspergillus nidulans* codon-adjusted mScarlet3 fluorescent protein, includes linker for N-terminal tagging.
pDM052	216814	*Aspergillus nidulans* codon-adjusted mNeonGreen fluorescent protein, includes linker for C-terminal tagging.
pDM053	216815	*Aspergillus nidulans* codon-adjusted mNeonGreen fluorescent protein, includes linker for N-terminal tagging.
pDM070	216816	*Aspergillus nidulans* codon-adjusted mCardinal fluorescent protein, includes linker for C-terminal tagging.
pDM071	216817	*Aspergillus nidulans* codon-adjusted mCardinal fluorescent protein, includes linker for N-terminal tagging.
pDM098	216818	*Aspergillus nidulans* codon-adjusted mStayGold fluorescent protein, includes linker for C-terminal tagging.
pDM097	216819	Aspergillus nidulans codon-adjusted mStayGold fluorescent protein, includes linker for N-terminal or internal tagging.
pDM067	216820	*Aspergillus nidulans* codon-adjusted mStayGold2 fluorescent protein, includes linker for C-terminal tagging.
pDM069	216821	*Aspergillus nidulans* codon-adjusted mStayGold2 fluorescent protein, includes linker for N-terminal tagging.

We chose to modify the system of Nødvig *et al.* ( Nødvig *et al.*, 2018; Nødvig *et al.*, 2015) because of its wide use in the field. Our system retains key elements of the Nødvig *et al*. ( Nødvig *et al.*, 2018; Nødvig *et al.*, 2015) system for fungal plasmid replication and expression of Cas9, sgRNA, and selectable markers. Because the Nødvig *et al.* system has been shown to work in multiple filamentous fungi ( Erdmann *et al.*, 2022; Mattos *et al.*, 2020; Nødvig *et al.*, 2018; Nødvig *et al.*, 2015; Schuster & Kahmann, 2019; Wenderoth *et al.*, 2017), we predict our system will work equally well in other fungal species.

We note that other CRISPR-Cas9 systems for *A. nidulans* and related fungi are also available ( Chang, 2023; Rozhkova & Kislitsin, 2021; Schuster & Kahmann, 2019; Shen *et al.*, 2023; Song *et al.*, 2019; Wang & Coleman, 2019; Yuan *et al.*, 2024). Importantly, the specific features of different systems can be either advantageous or disadvantageous, depending on the goals of the experiment, and thus it is important to consider the relevant trade-offs.

For example, *Aspergillus* CRISPR plasmids can be assembled using parts of a modular cloning-based synthetic biology toolkit that has a wide range of uses in filamentous fungi ( Mózsik *et al.*, 2021). While this approach offers considerable power and flexibility, it should be noted that plasmid construction via modular cloning normally requires two rounds of cloning: in the first round, the sgRNA transcriptional unit is assembled, and in the second round, the final plasmid carrying the sgRNA, Cas9 and a selectable marker is constructed. Therefore, for a simple task such as introducing one sgRNA sequence into a Cas9-expressing plasmid, a modular cloning approach may not be time- or cost-effective. Using this modular-cloning toolkit, a plasmid for one-step sgRNA cloning has been constructed, but for selection in *Aspergillus* this is currently limited to the *ble* (phleomycin-resistance) gene ( Mózsik *et al.*, 2021).

 Concurrent with our work, Yuan *et al.* adapted the Mortensen lab CRISPR plasmids for Golden Gate assembly ( Yuan *et al.*, 2024). Unlike our system, which uses annealed oligonucleotides to introduce an sgRNA spacer sequence into a CRISPR-Cas9 vector, the Yuan *et al.* system uses sgRNA units made from synthetic gene fragments and is specifically designed to facilitate cloning of a tRNA-gRNA array into a plasmid expressing Cas9 (or Cas12a) ( Yuan *et al.*, 2024). Having multiple sgRNAs, leading to multiple DSBs within a genomic region, may be particularly useful for making large genomic deletions (e.g. tens of kilobases), especially in contexts that do not require the precise editing offered by an introduced HR template ( Yuan *et al.*, 2024). Nødvig *et al.* also assembled multiple sgRNAs in a single CRISPR plasmid with their USER-based cloning system, in order to target multiple sites ( Nødvig *et al.*, 2018). However, the use of multiple sgRNAs may also increase the possibility of off-target effects, and thus may represent “overkill” if only a single DSB is required for a gene-editing experiment. In the context of precise, HR template-dependent genome editing and gene tagging at single loci, where a single sgRNA is often sufficient, the speed, efficiency, and low cost of our system may be particularly advantageous. For simultaneous targeting of two or more loci, our system could be modified to incorporate multiple sgRNAs within a single plasmid; alternatively, multiple single-sgRNA plasmids containing different selectable markers could be used simultaneously.

### Technical considerations for using the CRISPR-Cas9 system

In our experience, successful CRISPR-Cas9-based genetic modification in *A. nidulans* can vary among and within loci, depending on sgRNA efficiency (Extended data Tables S1, S2). Because of this, we recommend that researchers test sgRNA efficiency via the Technique to Assess Protospacer Efficiency (TAPE) assay described by Nødvig *et al.* ( Nødvig *et al.*, 2018), either prior to or simultaneous with a CRISPR-Cas9 editing experiment. TAPE takes advantage of the fact that homologous recombination experiments in *A. nidulans* are usually performed in strains carrying deletion of the *nkuA* gene, involved in NHEJ ( Nayak *et al.*, 2006). In TAPE, cells (i.e. *nkuA* mutants) are transformed with a CRISPR-Cas9 plasmid containing the relevant sgRNA but in the absence of an accompanying HR template. If an sgRNA efficiently promotes DSB formation, then cells will fail to form colonies on plates selective for the CRISPR-Cas9 plasmid, due to the combined absence of NHEJ and HR template-driven homologous recombination. By contrast, if an sgRNA does not promote DSB formation, then cells will be able to form colonies. When an sgRNA is found to be efficient via TAPE assay, transformation with a CRISPR-Cas9 plasmid together with a HR template leads to the vast majority of colonies (on selective plates) having the intended genetic modification (Extended data Tables S1, S2) ( Nødvig *et al.*, 2018). In this context, the ease with which our method enables cloning of sgRNA spacer sequences into CRISPR-Cas9 plasmids makes it possible to generate and test a wide variety of sgRNA spacer sequences in a short time.

With efficient sgRNAs, an edited or tagged strain can be generated in 7–10 days using our system (see Protocol). In particular, isolating individual strains by spore dissection rather than by multiple rounds of streak-purification on plates can save several days in the verification process, while also ensuring that selected strains are fully clonal. We used this approach to create a doubly-tagged strain by two successive transformations. Overall, when using efficient sgRNAs, the total time required to make two successive genetic modifications in a strain is approximately the same as the time required for a single genetic cross ( Todd *et al.*, 2007b).

We verified that our method can be used for gene editing via either single-stranded or double-stranded HR templates ( Figure 2, Extended data Table S1). Although a single-stranded HR template can be used, we would recommend using a double-stranded HR template, as it may be less likely to produce unintended mutations in the insertion locus ( Gallagher *et al.*, 2020).

### Recommendations for tagging with fluorescent proteins

We have used our CRISPR-Cas9 system to tag several proteins with *A. nidulans* codon-adjusted versions of recent-generation FPs ( Figure 3A, Extended data Table S2). The FP sequences include linkers for N- or C-terminal tagging ( Table 2; Methods). These will be useful reagents for *A. nidulans* cell biology research.

By tagging chitin synthase ChsB with five different FPs, we were able to evaluate the strengths and weaknesses of different FPs in *A. nidulans*. The highly photostable FPs mStayGold and mStayGold2 were of particular interest, as they have the potential to significantly enhance long-term imaging experiments ( Ando *et al.*, 2024). These proteins have been developed only recently and have not been thoroughly characterised in fungi. Surprisingly, we found that mStayGold is ten times less bright than mNeonGreen, at least when fused to the N-terminus of ChsB in *A. nidulans* ( Figure 3B). In mammalian cells, mStayGold has been shown to be as bright as, or brighter than, mNeonGreen ( Ando *et al.*, 2024). It is possible that the brightness of mStayGold depends on the local cellular environment or the context of the protein fusion. Further experiments tagging other proteins will be needed to determine whether mStayGold is consistently less bright than mNeonGreen in *A. nidulans* or other fungi. Other monomeric versions of StayGold have also been developed ( Ivorra-Molla *et al.*, 2024; Zhang *et al.*, 2024), and it is possible that these could have properties distinct from mStayGold when expressed in fungal cells. In any case, the mStayGold that we tested appears to be more photostable than mNeonGreen *in vivo* in *A. nidulans* ( Figure 3C) and thus may be a good choice for live-cell protein visualisation if the tagged protein is sufficiently abundant to yield a detectable fluorescent signal.

 In our view, mStayGold should be preferred over mStayGold2. mStayGold and mStayGold2 were initially described as equally bright and photostable, with mStayGold2 possibly better-suited for N-terminal tagging of proteins of interest ( Ando *et al.*, 2023). However, additional experiments by the same authors showed that mStayGold2 is slightly less photostable than mStayGold, and that mStayGold is in fact suitable for N-terminal tagging ( Ando *et al.*, 2024). Therefore, we would recommend the use of mStayGold over mStayGold2 for future gene tagging in *A. nidulans*.

Finally, we also note that the far-red fluorescent protein mCardinal has been reported to be a dimer *in vitro* ( Wannier *et al.*, 2018). As this could potentially perturb localization of a tagged protein, care should be taken when tagging with mCardinal.

Overall, the palette of fluorescent proteins presented here provides multiple options for cell biology experiments in *Aspergilli*.

## Methods

### Protocol

A detailed protocol of CRISPR plasmid construction and *A. nidulans* engineering can be found on protocols.io: Genetic engineering of *A. nidulans* using CRISPR-Cas9. DOI: dx.doi.org/10.17504/protocols.io.6qpvr3z93vmk/v1.

### Codon optimisation of fluorescent proteins

To generate a codon-usage table for *A. nidulans*, the coding sequences of *A. nidulans* genes with the assigned gene ontology term GO:0003735 ‘structural constituent of ribosome’ were downloaded from FungiDB ( https://fungidb.org) ( Basenko *et al.*, 2018). The sequences were then used to generate a codon-usage table using cusp ( https://www.bioinformatics.nl/cgi-bin/emboss/cusp). The generated codon usage table can be found in the Zenodo repository (Extended data Table S3). *A. nidulans* codon-adjusted gene sequences were generated using the python package DnaChisel ( https://edinburgh-genome-foundry.github.io/DnaChisel/) ( Zulkower & Rosser, 2020).

### Plasmid construction

Plasmid pDM026 (CRISPR-Cas9 vector with *pyrG* gene), pDM028 (CRISPR-Cas9 vector with *hph* gene), and pDM030 (CRISPR-Cas9 vector with *ble* gene) were constructed by respectively digesting pFC330 (Addgene #87842), pFC332 (Addgene #87845), and pFC333 (Addgene #87844) with restriction enzymes PacI (New England Biolabs, USA; #R0547) and BglII (New England Biolabs, USA; #R0144) and combining them with synthesized gene fragment gDM002 (Integrated DNA Technologies, USA) containing *A. nidulans* U3 promoter, HDV ribozyme, GFP drop-out cassette, glycine tRNA and U3 terminator via Gibson assembly ( Gibson *et al.*, 2009). Correct insertions in the final plasmids were verified by Sanger sequencing (Azenta, USA or MRC PPU DNA Sequencing and Services, UK).

Plasmid pDM068 (CRISPR-Cas9 vector with *NAT* gene), was constructed by replacing *pyrG* coding sequence with a *NAT* coding sequence via PCR amplification of pDM026, followed by Gibson assembly ( Gibson *et al.*, 2009). The *NAT* gene is therefore driven by the *A. fumigatus* pyrG promoter. The plasmid was verified by whole-plasmid sequencing (SNPsaurus, USA).

Plasmids containing *A. nidulans* codon-adjusted fluorescent proteins were constructed via Gibson assembly ( Gibson *et al.*, 2009), inserting synthesised gene fragments (Integrated DNA Technologies, USA) into pUC19 cloning vector. Regions of interest were sequenced by Sanger sequencing (Azenta, USA or MRC PPU DNA Sequencing and Services, UK).

CRISPR-Cas9 plasmids containing sgRNA spacer sequences were assembled by Golden Gate Reaction using PaqCI (#R0745, New England Biolabs, USA). 60bp-long complementary oligonucleotides were annealed by mixing 2.5 µL of each 100 µM oligo with 5 µL of annealing buffer (100 mM NaCl, 25 mM Tris, pH 7.5) in a 200 µL PCR tube. Forward and reverse oligonucleotide sequences were:

- Forward: 5’-ctaga cacctgcagcgggacNNNNNNNNNNNNNNNNNNNNgtttcgag gcaggtgcttcc-3'
  ◦ where “NNN…NNN” is the spacer sequence of the desired sgRNA. PaqCI recognition sites are underlined.
- Reverse: 5’-ggaag cacctgcctcgaaac *NNNNNNNNNNNNNNNNNNNN*gtcccgct gcaggtgtctag-3'
  ◦ where “ *NNN…NNN*” is the reverse complement of the spacer sequence of the desired sgRNA. PaqCI recognition sites are underlined.

The tube was placed in a thermocycler and incubated at 95°C for 3 minutes, followed by a ramp-down of 1°C every 30 seconds for 70 cycles (i.e. until reaching 25°C). The annealed oligonucleotide mix was then diluted 1:1750 in water.

The PaqCI Golden Gate reaction was set up in a 200µL PCR tube, using:

- 0.5 µl CRISPR-Cas9 vector plasmid (from 75 ng/µL stock).
- 0.5 µl diluted annealed spacer oligonucleotides.
- 0.25 µL PaqCI (New England Biolabs, USA; #R0745).
- 0.125 µL PaqCI activator (New England Biolabs, USA; #S0532SVIAL).
- 0.25 µL T4 Ligase (New England Biolabs, USA; #M0202).
- 1 µL T4 Ligase buffer (New England Biolabs, USA; #B0202SVIAL).
- 7.375 µL deionized water.

The reaction was placed in a thermocycler at 37°C for 60 minutes followed by 60°C for 5 minutes (if the reaction was then to be stored at -20°C, the 60°C incubation was repeated after thawing). For bacterial transformations, 30 µL of DH5α *E. coli* chemically competent cells were transformed with 2.5 µL of the Golden Gate reaction. After DNA minipreps, correct spacer insertion was verified by Sanger sequencing (Azenta, USA or MRC PPU DNA Sequencing and Services, UK), using primer oDM154.

Lists of all plasmids (including summaries of fluorescent protein amino-acid linker sequences), oligonucleotides, and gene fragments used in this study (Extended data Tables S4–S6) can be found in the Zenodo repository. Plasmid and gene-fragment DNA sequence files can also be found in the Zenodo repository.

### *A. nidulans* cultivation and strain construction

*A. nidulans* was grown on YG (20 g/L glucose, 5 g/L yeast extract, 1 mL/L Hutner’s trace element solution ( Hill & Kafer, 2001)) with supplements as required (2.44 g/L uridine, 1.01 g/L uracil, 0.411 mg/L pyridoxine). When grown on solid media, agar was added to a final concentration of 1.5%. Strains were incubated at either 30°C or 37°C.

For *A. nidulans* transformation *,* protoplasts were produced essentially as described in Oakley *et al.* ( Oakley *et al.*, 2012), but with the following modifications: 1) for cell-wall digestion, Extralyse ^®^ (Laffort, France) was used instead of VinoTaste ^®^ Pro; 2) all protoplast washes were performed at 4°C; and 3) Eppendorf Protein LoBind ^®^ tubes (Eppendorf, Germany; #0030108116) were used during protoplast washes to maximize protoplast recovery. All details of transformation are available as part of the protocol linked to this paper ( dx.doi.org/10.17504/protocols.io.6qpvr3z93vmk/v1). HR templates were generated by PCR (for gene tagging) or by annealing or annealing/extension of oligonucleotides (for gene editing; see Extended data Tables S1, S2, S5). Protoplasts were transformed with 1–2 µg of sgRNA plasmid and 0.5–1.5 µg of HR template. Transformations were plated on YG agar adjusted for osmolarity by having either final concentrations of 0.6 M KCl or 1 M sucrose. Nourseothricin was used at a final concentration of 150 µg/mL for selection of *NAT* resistance plasmids.

Transformation plates were incubated at 30°C for 1 day and 37°C for 3–4 days, until sporulating colonies formed. Strains were purified by dissection of single spores on a non-selective plate using a Singer MSM 400 Tetrad Dissection Microscope (Singer, USA).

Strains were verified by amplification of the genomic region of interest from 1–10 µL of a spore suspension first boiled at 95°C for 15 minutes and frozen at -70°C for 10 minutes, as described by Fraczek *et al.* ( Fraczek *et al.*, 2019). Genotyping PCRs were performed using NEB LongAmp Taq (New England Biolabs, USA; #M0323S) as in Fraczek *et al.* ( Fraczek *et al.*, 2019). High-fidelity spore PCRs were performed using Platinum™ SuperFi II DNA Polymerase (Thermo Fisher Scientific, USA; #12361010) and verified by Sanger sequencing (Azenta, USA or MRC PPU DNA Sequencing and Services, UK). All spore PCR methods are available as part of the protocol linked to this paper ( dx.doi.org/10.17504/protocols.io.6qpvr3z93vmk/v1).

A list of all *A. nidulans* strains used in this study (Extended data Table S7) can be found in the Zenodo repository. DNA sequence and sequencing files of edited and tagged loci can also be found in the Zenodo repository.

### Fluorescence microscopy

Spinning disk confocal microscopy ( Figure 3A–C) was performed using a Nikon Ti2-E microscope equipped with a Yokogawa CSU-W1 confocal scanner unit with a tri-band dichroic mirror (Di01-T405/488/561; Semrock), Plan Apo VC (100x/1.4 NA) oil objective and a Photometrics Prime 95B camera. Spores of relevant *A. nidulans* strains were inoculated on minimal media ( Hill & Kafer, 2001) agar plates with required supplements and grown for 16–20 hours at 30°C. Agar plugs containing hyphae were cut from the plate and inverted onto Ibidi 4-well µ-Slide glass-bottom chambers (Cat. No: 80427, Ibidi GmbH, Germany) filled with 200 µL liquid minimal media with supplements. To allow cells to adapt to the imaging temperature (18–20°C), chambers with cells were left on the microscope stage for at least 30 minutes before the start of imaging. For imaging NeonGreen, mStayGold and mStayGold2, a 488 nm laser was used for excitation, with a 525/50 nm band-pass emission filter. For mScarlet3 and mCardinal, a 561 nm laser was for excitation, with a 568 nm long-pass emission filter. For each image, 21 Z-sections were acquired, with 0.2 µm spacing. Z-position was controlled using a NIDAQ Piezo Z device. Imaging of time-courses ( Figure 3C) used a 70 ms exposure per Z-section, with continuous illumination and acquisition until the fluorescent signal was no longer detected.

Laser scanning confocal microscopy ( Figure 3D) was performed using a Zeiss LSM 980 microscope equipped with an Airyscan 2 detector. On the day before imaging, 10 ^3^ and 10 ^4^ spores (to provide different cell densities at the time of imaging) of strain aDM029 were inoculated in 400 µL of liquid minimal medium ( Hill & Kafer, 2001) with required supplements in Ibidi 8-well µ-Slide glass-bottom chambers (Cat. No: 80827, Ibidi GmbH, Germany) and grown at 30°C overnight. Imaging was done at 37°C in a temperature-controlled environmental chamber. To allow cells to adapt to the imaging temperature, chambers with cells with cells were left on the microscope stage for at least 30 minutes before the start of imaging. Cells were imaged using an Alpha Plan Apochromat (x100/1.45 NA) oil objective and a 488/561 dual-band dichroic mirror. mScarlet3-chsB and mStayGold2-fksA were excited using 561 nm and 488 nm lasers respectively. 29 Z-sections (0.14 µm spacing) were acquired using Airyscan 2 Multiplex mode; both channels were imaged sequentially in the same Z-section before the next Z-section was imaged. Images were deconvolved using Huygens Software ( https://svi.nl/Huygens-Software). FIJI ( Schindelin *et al.*, 2012), is a free alternative that can perform deconvolution.

All microscopy images were processed using FIJI ( Schindelin *et al.*, 2012). Figures were assembled using Adobe illustrator ( https://www.adobe.com/products/illustrator.html). Inkscape ( https://inkscape.org) is a free alternative that can be used for figure assembly.

## Ethics and consent statement

Ethical approval and consent were not required.

## Data Availability

Zenodo: Improved gene editing and fluorescent-protein tagging in Aspergillus nidulans using a Golden Gate-based CRISPR-Cas9 plasmid system. https://doi.org/10.5281/zenodo.10700141 ( Modaffari, 2024). The project contains the following underlying data: DNA_files.zip. ZIP file containing annotated sequence information for all plasmids, gene fragments and *A. nidulans* genetic loci used in this work, presented in .gbk format. In addition, sequencing files for *A. nidulans* strain verification are presented in .ab1 or .fasta format. All formats can be opened using free software ApE ( https://jorgensen.biology.utah.edu/wayned/ape/) code.zip. ZIP file containing python script for *A. nidulans* codon-adjusting and R script for generating graph and statistical test of Figure 3B, including tip measurement tables for Figure 3B. microscopy_data.zip. ZIP file containing raw microscopy data files for all biological images, in either .tif, .nd2, or .czi format. All formats can be opened using free open-source software FIJI ( Schindelin *et al.*, 2012). README.txt. Text file describing the structure of the repository. Zenodo: Improved gene editing and fluorescent-protein tagging in Aspergillus nidulans using a Golden Gate-based CRISPR-Cas9 plasmid system. https://doi.org/10.5281/zenodo.10700141 ( Modaffari, 2024). The project contains the following extended data: combined_extended_data.pdf. All extended data supplementary figures and tables. extended_data_tables.zip. ZIP file containing separate supplementary data tables in .pdf or .csv format. extended_data_figures.zip. ZIP file containing extended data supplementary figures in .pdf format. README.txt. Text file describing the structure of the repository. Data are available under the terms of the Creative Commons Zero “No rights reserved” data waiver (CC0 1.0 Public domain dedication).

## Software availability statement

Archived scripts used in this publication are available from: https://doi.org/10.5281/zenodo.10700141.
