# Supplementary material for: Improved gene editing and fluorescent-protein tagging in Aspergillus nidulans using a Golden Gate-based CRISPR-Cas9 plasmid system
Source: Wellcome Open Res. 2024 Oct 17;9:602. [Version 1] doi: 10.12688/wellcomeopenres.23086.1 (PMC11617824; doi:10.12688/wellcomeopenres.23086.1)
Supplement: Peer review history

# Reviewer report: Reviewer response for version 1

**DOI:** 10.21956/wellcomeopenres.25422.r112747

**Published:** 2024-12-04

**Authors:** Reinhard Fischer

**Affiliations:** 1 Karlsruhe Institute of Technology (KIT), Karlsruhe, Germany

The paper by Modaffari et al. describes the improvement of a vector system for CRISPR Cas9 genome editing in fungi, published by Nodvig et al. in 2018. There were several improvements which are useful for the application. In addition they describe new fluorescent proteins, which is also very useful for the fungal community. The system could be further developed to easily label fungal organelles as described 20 years ago (Toews, M.W., Warmbold, J., Konzack, S., Rischitor, E.P., Veith, D., Vinuesa, C., Vienken, K., Wei, H. & Fischer, R. (2004) Establishment of mRFP1 as a fluorescent marker in *Aspergillus nidulans* and construction of expression vectors for high-throughput protein tagging using recombination in vitro (GATEWAY). Curr. Genet., 45:883-889.). This could be extended to peroxisomes, endosomes and others. I suggest abbreviating Aspergillus nidulans after the first use.

Is the rationale for developing the new method (or application) clearly explained?

Yes

Is the description of the method technically sound?

Yes

Are the conclusions about the method and its performance adequately supported by the findings presented in the article?

Yes

If any results are presented, are all the source data underlying the results available to ensure full reproducibility?

Yes

Are sufficient details provided to allow replication of the method development and its use by others?

Yes

Reviewer Expertise:

Fungal Genetics and Fungal Cell Biology

I confirm that I have read this submission and believe that I have an appropriate level of expertise to confirm that it is of an acceptable scientific standard.

# Reviewer report: Reviewer response for version 1

**DOI:** 10.21956/wellcomeopenres.25422.r107520

**Published:** 2024-11-01

**Authors:** HeeSoo Park

**Affiliations:** 1 Department of Molecular Genetics and Microbiology, Kyungpook National University, Durham, NC, 27710, South Korea

In this manuscript, the authors construct a Golden Gate-based sgRNA plasmid to improve the CRISPR-Cas9 plasmid system. This plasmid system allows us to perform the transformation process in E. coli very efficiently. The authors demonstrated this process using the  Golden Gate-based sgRNA plasmid system in Aspergillus nidulans. Overall, this system can greatly improve the gene editing system in A. nidulans.

Is the rationale for developing the new method (or application) clearly explained?

Yes

Is the description of the method technically sound?

Yes

Are the conclusions about the method and its performance adequately supported by the findings presented in the article?

Yes

If any results are presented, are all the source data underlying the results available to ensure full reproducibility?

Yes

Are sufficient details provided to allow replication of the method development and its use by others?

Yes

Reviewer Expertise:

Fungal genetics

I confirm that I have read this submission and believe that I have an appropriate level of expertise to confirm that it is of an acceptable scientific standard.
